# Novel Cooperative Scheme Based on Joint Band Assignment and Power Allocation for a Coexisting Radar-Communications System

**DOI:** 10.3390/s21186062

**Published:** 2021-09-10

**Authors:** Yufeng Chen, Guisheng Liao, Zhiwei Yang, Yongjun Liu, Mengchao Jiang

**Affiliations:** National Laboratory of Radar Signal Processing, Xidian University, Xi’an 710071, China; liaogs@xidian.edu.cn (G.L.); yangzw@xidian.edu.cn (Z.Y.); yjliuinsist@163.com (Y.L.); chinaexjmc@163.com (M.J.)

**Keywords:** cooperative scheme, joint optimization, coexisting radar-communications (CRC) system, band assignment, power allocation, spectral estimation

## Abstract

In this paper, we present a novel cooperative scheme of joint optimal resource allocation, such that the overall performance of the coexisting radar-communications (CRC) system can be improved. In our proposed scheme, target detection and multiuser communication are performed by radar and communication subsystems at the same time, as well as a control center, which is responsible for joint resource management. We aim to minimize the ISLR for target detection and maximize the sum-rate for communications simultaneously by jointly optimizing the band assignment and transmit power allocation. Since the resulting optimization problem involving two performance metrics and a binary constraint is a multiobjective nonconvex problem, a two-tier iterative decomposition (TT-ID) approach is devised to obtain the globally optimal solution. However, compared with the conventional radar signals, the autocorrelation function of the devised radar signal may still have relatively high sidelobes. In particular, when the data transmission becomes the primary purpose of the CRC system, the sidelobe performance gets worse. As a consequence, some weak targets are most likely overshadowed by the adjacent strong targets through the matched filtering at the radar receiver. To address this, a spectral estimation algorithm based on the Bayes Cauchy–Gaussian (Bayes–CG) model is employed to further reduce the range sidelobes of the matched filter output at the radar receiver according to the prior distribution of the desired autocorrelation. Finally, several numerical results are provided to show the merits of the proposed method.

## 1. Introduction

Owing to the rapid growth of electronic devices, the electromagnetic frequency spectrum is increasingly congested. As a consequence, radio frequency interference (RFI) is a sharp challenge to face. It is shown that RFI significantly deteriorates the performance for radar and communication applications. Recently, the design of a coexisting radar-communications (CRC) system is becoming a key mechanism to cope with this challenge, which has been widely used in several scenarios, such as the spectral coexistence of airborne early warning radar systems and time division duplexing long term evolution (TDD-LTE) systems [1], spectrum sharing between air traffic radars and commercial cellular networks or wireless local area networks (WLAN) [2], and internet of vehicles [3]. It is shown that the CRC techniques can enable radar and communication systems to share the same bandwidth by mitigating mutual interference through some common protocols and strategies.

Technically speaking, the signaling strategy of the CRC system includes three main categories, namely time-division based design, frequency-division based design, and spatial-division based design [4]. A straightforward time-division method uses a switch to allocate different time slots to the radar and communication functions, which can be found in autonomous vehicle applications [5]. The studies in [6,7] indicate that the preamble duration increments are potential solutions to improve the detection range of the radar but with the trade-off of communication performance. Instead, a cycle time frame is proposed in [8], where the time portion for any function in the current cycle time can vary adaptively to improve the radar estimate rate and communication rate in the meantime. The study in [9] investigates the frequency division-based technique in radar-communication spectral coexistence for the first time, where a spectral null is added to the ultra-wide band (UWD) noise radar waveform on purpose, and the orthogonal frequency division multiplexing (OFDM) signal for communication can then be embedded in above-mentioned notch. Inspired by this work, the design of a spectrally compliant waveform has been widely exploited in [10,11,12,13]. Similarly, the authors in [14,15] have so far investigated the frequency-modulated continuous wave (FMCW). As spatial approaches, the key idea of [16,17] is to project the radar waveform onto the null space of the interference channel from the radar transmitter to the communication receiver. Moreover, the works in [18,19] have proved that a lower effective interference power (EIP) can be obtained by a joint radar sampling and communication precoding matrix design under the constraints of the transmit power budget and target communication capacity. However, the above studies just focus on mitigating the mutual interference between radar and communication subsystems without regards to several arising challenges, such as performance degradation, low resource utilization, and complicated design and processing.

Motivated by the great demand for performance improvement and resource management in CRC systems, a cooperative design has attracted substantial attention for recent years. Unlike the conventional noncooperative systems, the cooperative schemes aim to share the information and adaptively allocate the resources between the subsystems so as to enhance the performance of the radar and communication functions [20]. For example, a cooperative CRC system is developed in [21], where a water-filling successive interference cancellation (SIC) technique is employed at the joint radar-communications receiver to maximize the data rate. Meanwhile, a unimodular radar waveform is designed to guarantee fine correlation performance by using a spectral shape method. With the objective of minimizing the transmit beamforming error subject to the total power constraint and per-antenna power constraint, the works in [22,23] also consider the transmit covariance matrices for two functions in different array deployments, which is inspired by the nullspace projection method [16,17]. Specially, the contribution in [23] further improves the spatial gain through an appropriate beampattern template and an interleaved array optimization. Additionally, several sparse array configurations are developed in [24,25], where two simultaneous beamformers for different functions are implemented by antenna selection. On the other hand, the works in [26,27,28,29,30,31,32,33,34] target power allocation strategies to improve the spectrum efficiency of the CRC system. In [26], the authors formulate a problem to maximize the radar SINR with the constraints of transmit power and downlink communication rate. Later on, the works in [27,28] focus on multiuser communication scenarios. Since the conditional mutual information (MI) can be measured by the accuracy of target estimation for radar detection, the purpose of [27] is to maximize the weighted summation of MI for radar and communication purposes by optimizing the covariance matrices of transmit waveforms. As an extension, the studies in [28] consider the transmit beampattern design in perfect and imperfect channel state information (CSI) cases. Similarly, joint designs of communication code-book and radar waveform/received filter are developed to maximize the SINR and MI in [29,30,31], respectively. Different from the aforementioned works, the authors in [32] study a robust OFDM waveform optimization in signal-dependent clutters under the condition of uncertainty target spectra, whereas the communication signals backscattered off the targets at the radar receiver can be ignored or viewed as interference and even useful energy based on the prior knowledge of the communication signals, the target spectra, and the propagation loss of the channels. In addition, a low probability of intercept (LPI)-based waveform optimization for the CRC system in the same scenario is taken into account in [33]. These authors further present a novel optimal power allocation for a bistatic CRC system in [34], whose goal is to achieve the prescribed data rate for the communication receiver and LPI performance for the radar receiver by minimizing the transmit power consumption for both communication symbols and radar waveform.

In this paper, we focus on the joint design of a frequency-division-based approach for a CRC system. It is well-known that this approach has the advantage of simplicity and ease of implementation, existing, however, with several flaws, including low spectrum efficiency and performance loss. To tackle these issues, we develop a cooperative scheme in which the radar and communication performances can be enhanced simultaneously by jointly allocating the bandwidth and transmit power resources on the radar subsystem and communication subsystem. To this end, a control center is devised to implement the information sharing between the above two subsystems and subsequently realize the optimal resource management according to the practical demands.

For clarity, our contributions are summarized as follows:

(1) We present a novel cooperative scheme for a frequency-division-based approach and establish the specific resource model in a CRC system. In our proposed scheme, a control center is equipped to collect the necessary information from the radar and communication subsystems, solve the joint resource allocation problem, and assign the corresponding optimal parameters to each subsystem. In order to assess the performance metrics of two functions well, two resource allocation models are established through a two-scale approach; one called the rough scale resource allocation for communications, and another one called the thinning scale resource allocation for radar.
(2) For the sake of enhancing the detection performance and transmission capacity of the CRC system simultaneously, a joint working band assignment and transmit power allocation strategy is investigated. Different from the above-mentioned works, we focus on optimizing the range sidelobe level and sum-rate subject to several resource constraints, which are important performance metrics for target detection and multiuser communication networks, respectively.
(3) A global optimization algorithm based on the heuristic method and decomposition for solving the aforementioned problem is developed. Due to the performance metrics and binary constraint, the joint resource allocation is a multiobjective nonconvex problem and NP-hard. Based on the relationship between the two types of resource allocation variables, we develop a two-tier solution methodology, where the original nonconvex problem can be decomposed into two subproblems. We first search the optimal band selection variable with fixed power allocation variables. For a given band selection variable, the power allocation subproblem can be further divided into two convex sub-subproblems, which both can be solvable by the Lagrange multiplier approach.
(4) Due to the disjoint spectrum, the autocorrelation sidelobes of the radar signal obtained by joint resource allocation probably still reach an unsatisfying level compared with the conventional radar signals, especially when the sum-rate performance is the top priority of the CRC system. In such cases, the matched filtering procedure in the radar receiver fails to detect some weak targets that are overshadowed by the nearby strong targets. Since the matched filtering model with respect to the radar signal can be viewed as a missing data recovery problem in the frequency domain, the high sidelobes of the matched filter output can be effectively suppressed by the spectral estimation algorithm based on the prior knowledge of the desired autocorrelation response.

To the best of our knowledge, the joint resource allocation scheme for range sidelobe minimization for probing waveform and the sum-data maximization for multiuser communication network has not been exploited in studies on the design of frequency-division-based CRC system. The proposed optimization leads to a high computational complexity. Nevertheless, this challenge can be effectively tackled by the equipped control center thanks to the ever-growing cloud computing techniques and specific integrated circuit designs.

The rest of this paper is organized as follows. In Section 2, we introduce the devised cooperative scheme as well as the system model of the CRC system. The ISLR for the radar metric and sum-rate for the communication metric are also formulated. In Section 3, the joint optimization subject to resource allocation is proposed. Section 4 provides an effective approach to further suppress the sidelobes in signal processing in the case that the autocorrelation of the devised radar signal still maintains an unacceptable sidelobe level. Several numerical results are provided to validate that the proposed method can improve the target detection performance and data transmission rate of the CRC system in Section 5. Finally, conclusions are drawn in Section 6.

**Notation** **1.** In this paper, bold lower-case letters and bold upper-case letters represent the vectors and matrices, respectively. R and C represent the real and complex set, respectively. * represents the convolution operator. *⊗* and *⊙*, respectively, represent Kronecker product and Hadamard product. ${\left(\cdot \right)}^{T}$, ${\left(\cdot \right)}^{*}$, and ${\left(\cdot \right)}^{H}$ represent the transpose, complex conjugate, and Hermitian transpose, respectively. ${\left(\cdot \right)}^{r}$ represents the reverse of the argument. ${∥\cdot ∥}_{2}$ represents the $l_{2}$ norm. $diag\left(\cdot \right)$ represents the diagonal matrix with the elements given by the vector arguments. $I_{\left(\cdot \right)}$ represents the prescribed-dimensionality identity matrix. $1_{\left(\cdot \right)}$ and $0_{\left(\cdot \right)}$ represent prescribed-length all-ones and all-zeros vector, respectively. $\left\lceil \cdot \right\rceil$ represents the round up of the argument.

## 2. System Model

**Figure 1:**
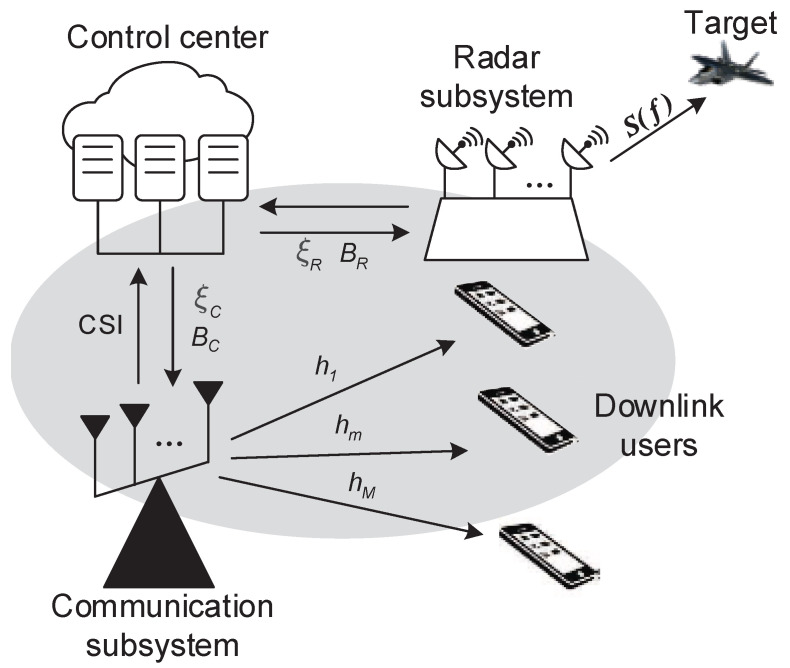
The proposed cooperative CRC system, including a control center, a radar subsystem, and a communication subsystem.

**Figure 2:**
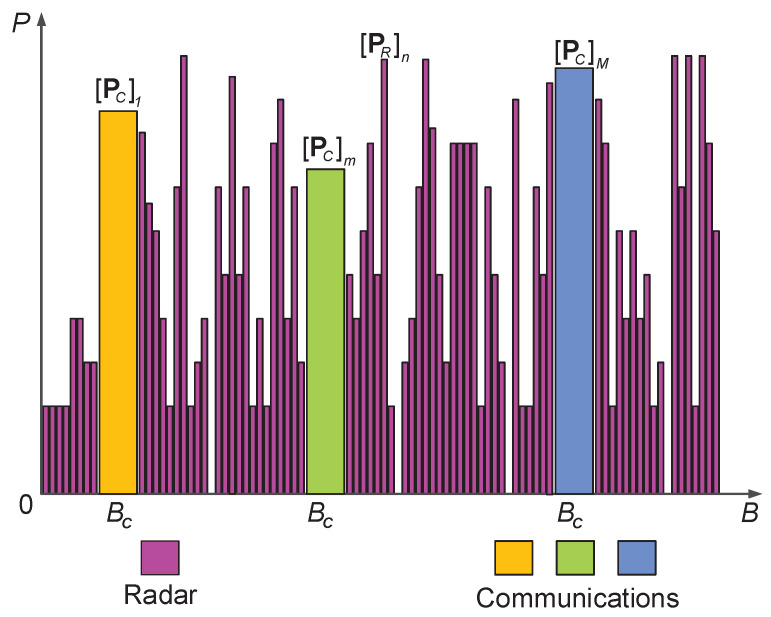
The resource model of different subsystems. ${\left[p_{C}\right]}_{m}$ and ${\left[p_{R}\right]}_{n}$ are the transmit powers assigned to the *m*-th communication symbol and allocated on the *n*-th frequency bin for the radar subsystem.

Let us consider a CRC system, which is equipped with a control center, a radar subsystem, and a communication subsystem, as illustrated in Figure 1. Except the basic dual functionality implemented by radar and communication subsystems in the meantime, a cooperative scheme for the CRC system is proposed, where the control center is designed to collect information from above two subsystems, solve joint optimization problems, and optimally allocate the transmit power and working band to each subsystem dynamically, according to the designer’s demands. Moreover, we rely on the following assumptions:

(1) The spectrally compliant waveform is transmitted to perform the target detection, while preventing the communication subsystem from the strong radio interference of high-power radar signals. Thus, the radar signal can be equivalently viewed as an FDM signal among the communication signals.
(2) The communication subsystem and *M* downlink users constitute a communication network together, in which *M* communication symbols are sent in parallel to serve corresponding downlink users via FDM technology. The communication symbols are occupying the same bandwidths (denoted by $B_{C}$), statistically independent of the radar signal, and arbitrary information modulations are allowed to use. Moreover, the communication channel is slow time variant and frequency selective fading, and CSI can be perfectly estimated by pilot symbols [28].
(3) The resources for radar use and communication use are, respectively, modeled from two scale viewpoints, as shown in Figure 2. The rough scale resource model is defined that radar and communication subsystem share the total transmit power resource $\xi_{t}$ and bandwidth resource *B*. In particular, *B* is an integral multiple of the bandwidth occupied by each communication signal. That is to say, it can be divided into *K* subband sections, *M* of which are assigned to the communication subsystem, while the rest are accessed by radar. In radar subsystem, the thinning transmit power allocated on frequency bins is considered, which is an underlying degree of freedom to achieve the desired performance. That is referred to as the thinning scale resource model.

Accordingly, the transmitted signal of the cooperative CRC system can be formulated in the frequency domain as

(1) $$\begin{array}{cc} s & =\left(e⊗1_{L}\right)⊙z+\left[\left(1_{K}-e\right)⊙c\right]⊗1_{L}\\ \; & =\left(e⊗1_{L}\right)⊙\left(\Phi_{R}⊙p_{R}^{1/2}\right)+\left[\left(1_{K}-e\right)⊙\left(\Phi_{C}⊙p_{C}^{1/2}\right)\right]⊗1_{L} \end{array}$$

where $z\in C^{N_{s}\times 1}$ is the frequency domain transmitted radar waveform, where $N_{s}$ is the sample length. The vector $c\in C^{K\times 1}$ is a set of communication signals in the frequency domain. *L* is the thinning frequency sampling factor with satisfying $L=N_{s}/K$. $p_{R}\in R^{N_{s}\times 1}$ and $p_{C}\in R^{K\times 1}$ stand for the power spectrum density (PSD) sequences of the radar waveform z and communication signals c, respectively. By introducing a phase auxiliary vector $\Phi_{R}\in C^{N_{s}\times 1}$, the radar waveform z can be decomposed into the amplitude component $p_{R}^{1/2}$ and the phase component $\Phi_{R}$. Similarly, $p_{C}^{1/2}$ and $\Phi_{C}\in C^{K\times 1}$ denote the envelope and phase information of the communication signal vector c, respectively. Since z and c are non-overlapping in the frequency domain, the vector $e\in R^{K\times 1}$ is used to select the working band sections that are assigned to the radar subsystem, which is defined by

(2) $$e_{k}=\left\{\begin{array}{c} 1,\;k\;\mathrm{belongs}\;\mathrm{to}\;\mathrm{the}\;\mathrm{radar}\;\mathrm{subsystem}\\ 0,\;\mathrm{otherwise} \end{array}\right.\;$$

According to Assumption (3) above, we define the bandwidth occupancy ratio of the radar subsystem as

(3) $$\eta =\frac{K-M}{K}$$

Likewise, we define the power occupancy ratio of the radar subsystem as

(4) $$\rho =\frac{\xi_{R}}{\xi_{t}}$$

where $\xi_{R}$ denotes the transmit power allocated to the radar subsystem.

### 2.1. Range Sidelobe Level for Radar

For target detection, matched filtering is a well-known technique to estimate the target information in the ranges of interest. It can be accomplished by convolving the radar return signal with the transmitted waveform, which indicates that the detection performance is determined by the autocorrelation performance of the transmitted waveform. In particular, low range sidelobes will prevent the weak targets from the interferences caused by the ambient strong targets [35]. To analyze this issue, we use the integrated sidelobe level ratio (ISLR) metric to measure the range sidelobe level, and it is defined as

(5) $$\mathrm{ISLR}\;=\frac{1}{r_{0}^{2}}\sum_{\tau \in \Theta_{s}}{\left|r_{\tau }\right|}^{2}$$

where $r={\left[r_{-N_{s}+1},\ldots ,r_{0},\ldots ,r_{N_{s}}\right]}^{T}$ denotes the autocorrelation function (ACF) sequence of the waveform, where $r_{0}$ is a constant representing the total energy. $\Theta_{s}=\left[-N_{s}+1,-\Delta \right)\cup \left(\Delta ,N_{s}\right]$ denotes the index set of the sidelobe region of the ACF, where Δ is the sample index of the autocorrelation mainlobe width. To facilitate the calculation, here, Δ is defined as the Nyquist distance [36], and the sample length of r is set to be $2N_{s}$.

Since the ACF and PSD form a Fourier transform pair, the ISLR can be also rewritten as

(6) $$\;\mathrm{ISLR}=\frac{1}{r_{0}^{2}}\sum_{\tau \in \Theta_{s}}{\left|f_{\tau }^{H}\left[\left(e⊗1_{L}\right)⊙p_{R}\right]\right|}^{2}$$

where $f_{\tau }\in C^{N_{s}\times 1}$ here denotes the τ-th column of the DFT matrix $F_{N_{s}}\in C^{N_{s}\times 2N_{s}}$, which is given by

(7) $${\left[F_{N_{s}}\right]}_{n,\tau }=\exp\left[-j2\pi \frac{\left(n-1\right)\left(\tau -1\right)}{N_{s}}\right],\;n=1,\ldots ,N_{s},\tau =1,\ldots ,2N_{s}$$

**Remark** **1.** *It is worthy to mention that the ultimate goal is to achieve a spectrally compliant waveform with the desired PSD (equivalently the ACF). Since the studies on PSD-to-waveform implementation approaches have been widely exploited in [10,11,12,13]; in this paper, we focus on optimizing the resource allocation to minimize the ISLR.*

### 2.2. Sum-Rate for Communication

As to the communications, the sum-rate is a significant performance metric in the multiuser network [37]. In the frequency selective fading channel, the transmit power assignment and channel quality both play important roles in the data rate for each communication link. Accordingly, in the devised cooperative scheme, the achievable sum-rate can be formulated in the following by employing the parameters defined previously in Equations (1) and (2)

(8) $$R_{t}=\sum_{k=1}^{K}B_{C}\log_{2}\left(1+\frac{{\left(1-e_{k}\right)\left|h_{k}\right|}^{2}{\left[p_{C}\right]}_{k}}{\sigma_{C}^{2}}\right)$$

where $h_{k}$ denotes the channel frequency response of the *m*-th communication symbols, and $\sigma_{C}$ is the noise power in the communications channel.

## 3. Joint Optimization for the CRC System

We devote this section to the joint working band assignment and transmit power allocation for the cooperative CRC system to maximize the weighted summation of radar and communication metrics. Then, a two-tier iteration solution is developed to solve the resources allocation problem.

### 3.1. Problem Formulation

As discussed in the last section, whether the ISLR or the sum-rate is heavily dependent on the working band selection variable e and transmit power allocation variables $\left\{p_{R},p_{C}\right\}.$ Therefore, our goal is to minimize the ISLR for target detection while maximizing the sum-rate for the multiuser network by jointly allocating the available bandwidth and power resources. Taking into account the limitations on those resources, the joint resource allocation problem for the cooperative CRC system is also subject to the following constraints.

(1) To maximize the probability of target detection, the transmit power $\xi_{R}$ is no doubt fully utilized in the radar subsystem. Thus, the following transmit power requirements should be met
  (9) $$\left\{\begin{array}{c} \sum_{n=1}^{N_{s}}{\left[\left(e⊗1_{L}\right)⊙p_{R}\right]}_{n}=\xi_{R}=\rho \xi_{t}\\ {\left[p_{R}\right]}_{n}\geq 0,\;n=1,\ldots ,N_{s} \end{array}\right.$$
(2) Our criterion for the multiuser network is to maximize the sum-rate while guaranteeing the data rate required for each user. Hence, the transmit power should be subject to the constraints as follows
  (10) $$\left\{\begin{array}{c} \log_{2}\left(1+\left(1-e_{k}\right)g_{k}{\left[p_{C}\right]}_{k}\right)\geq \zeta ,\;k=1,\ldots ,K\\ \sum_{k=1}^{K}\left(1-e_{k}\right){\left[p_{C}\right]}_{k}=\xi_{t}-\xi_{R}=\left(1-\rho \right)\xi_{t} \end{array}\right.$$
  where ζ is the specified data rate threshold for each user; $g_{k}={\left|h_{k}\right|}^{2}/\sigma_{C}^{2}$ for simplicity. The second constraint is determined by the conservation of energy.
(3) As previously defined in Equation (3), only K−M subbands can be allocated for radar use. Thus, for the binary type parameter $e_{k}$, the following constraints should be met
  (11) $$\left\{\begin{array}{c} \sum_{k=1}^{K}e_{k}=K-M=\eta K\\ e_{k}\in \left\{0,1\right\},\;i=1,\ldots ,K \end{array}\right.$$

Then, fusing the above-mentioned constraints together, the joint optimization problem for the cooperative CRC system with respect to the working band selection variable e and transmit power allocation variables $\left\{p_{R},p_{C}\right\}$ can be formulated as

(12a) $$\begin{array}{cc} \max_{e,p_{R},p_{C}} & \frac{\omega }{F_{R}}{\mathrm{ISLR}}^{-1}\left(e,p_{R}\right)+\frac{\left(1-\omega \right)}{F_{C}}R_{t}\left(e,p_{C}\right) \end{array}$$

(12b) $$\begin{array}{cc} s.t.\; & \sum_{n=1}^{N_{s}}{\left[\left(e⊗1_{L}\right)⊙p_{R}\right]}_{n}=\rho \xi_{t} \end{array}$$

(12c) $$\begin{array}{cc} \; & {\left[p_{R}\right]}_{n}\geq 0,\;n=1,\ldots ,N_{s} \end{array}$$

(12d) $$\begin{array}{cc} \; & \log_{2}\left(1+\left(1-e_{k}\right)g_{k}{\left[p_{C}\right]}_{k}\right)\geq \zeta ,\;k=1,\ldots ,K \end{array}$$

(12e) $$\begin{array}{cc} \; & \sum_{k=1}^{K}\left(1-e_{k}\right){\left[p_{C}\right]}_{k}=\left(1-\rho \right)\xi_{t} \end{array}$$

(12f) $$\begin{array}{cc} \; & \sum_{k=1}^{K}e_{k}=\eta K \end{array}$$

(12g) $$\begin{array}{cc} \; & e_{k}\in \left\{0,1\right\},\;k=1,\ldots ,K \end{array}$$

where ω is a weighting factor that determines the task priority of radar and communication, according to the practical demands. $F_{R}$ and $F_{C}$ are the optimal conditions for the radar and communication criteria under the constraints, respectively. Due to the two performance metrics involved in Equation (12a) and the binary constraint in Equation (12g), the optimization problem in Equation (12) is a multiobjective nonconvex mixed integer programming (MNMIP), and there is no efficient approach to solve this problem directly in polynomial time [37]. In what follows, we propose a two-tier iterative decomposition (TT-ID) algorithm to obtain the globally optimal solution of the optimization problem in Equation (12).

**Remark** **2.** *Instead of ISLR, we employ the reciprocal of ISLR as the first part of the objective function in Equation (12) for the more intuitive formulation, which can also be interpreted as the ratio of mainlobe energy to the integrated sidelobe energy based on the fact that the maximization of ${\mathrm{ISLR}}^{-1}$ is the dual-problem of the minimization of ISLR.*

### 3.2. Proposed Solution by TT-ID Algorithm

Notice that the transmit power allocation variables $\left\{p_{R},p_{C}\right\}$ are both coupled with the working band selection variable e in Equation (12). For this reason, we develop a two-tier iteration approach for eliminating the aforementioned couples. As a consequence, the original problem can be decomposed into a band assignment subproblem and a power allocation subproblem. For the given $p_{R}$ and $p_{C}$, we first aim to determine e by using the heuristic method, which is named the outer tier procedure for solving the band assignment subproblem. Then, we further partition the power allocation subproblem into two convex sub-subproblems, where $p_{R}$ and $p_{C}$ can be efficiently optimized with a fixed e. It is referred as to the inner tier procedure. The above procedures are executed iteratively until convergency.

Accordingly, with fixed transmit power allocation variables $\left\{p_{R},p_{C}\right\}$, the original problem can be reformulated as the following band assignment subproblem

(13a) $$\begin{array}{cc} \max_{e} & \frac{\omega }{F_{R}}{\mathrm{ISLR}}^{-1}\left(e\right)+\frac{\left(1-\omega \right)}{F_{C}}R_{t}\left(e\right) \end{array}$$

(13b) $$\begin{array}{cc} s.t. & \sum_{k=1}^{K}e_{k}=\eta K \end{array}$$

(13c) $$\begin{array}{c} e_{k}\in \left\{0,1\right\},\;k=1,\ldots ,K \end{array}$$

Likewise, the original problem can be rewritten as the following power allocation subproblem for the given working band selection variable e

(14) $$\begin{array}{cc} \max_{p_{R},p_{C}} & \;\frac{\omega }{F_{R}}{\mathrm{ISLR}}^{-1}\left(p_{R}\right)+\frac{\left(1-\omega \right)}{F_{C}}R_{t}\left(p_{C}\right)\\ s.t.\; & \sum_{n=1}^{N_{s}}{\left[\left(e⊗1_{L}\right)⊙p_{R}\right]}_{n}=\rho \xi_{t}\\ \; & {\left[p_{R}\right]}_{n}\geq 0,\;n=1,\ldots ,N_{s}\\ \; & \log_{2}\left(1+\left(1-e_{k}\right)g_{k}{\left[p_{C}\right]}_{k}\right)\geq \zeta ,\;k=1,\ldots ,K\\ \; & \sum_{k=1}^{K}\left(1-e_{k}\right){\left[p_{C}\right]}_{k}=\left(1-\rho \right)\xi_{t} \end{array}$$

#### 3.2.1. Outer Tier for Band Assignment

In the outer tier procedure, we focus on how to solve the band assignment subproblem (13). It can be seen that with the binary constraint of the band selection variable e, the problem in (13) is a typical 0-1 inter programming, which is the most intractable part to address. For this reason, a heuristic search based on the nondominated sorting genetic algorithm (NSGA-II) is employed to find the globally optimal solution.

It is well-known that NSGA-II is well suitable for solving multiobjective optimization problems because the optimal solution can be obtained by searching exhaustively in parallel, regardless of the convexity of the problem [38]. Besides, NSGA-II provides two major advantages of the lower computational complexity of the nondominated sorting procedure, and an elite preservation strategy introduced to prevent good solutions from the spread of solution regions, compared to the conventional heuristic methods. The outer tier for optimal band assignment using NSGA-II with some modifications is introduced in the following.

At the beginning of NSGA-II, a random set of *P* individuals of the working band selection variable e is initialized, with meeting the constraint condition in Equation (11). The *p*-th individual for the *q*-th generation is defined as $e^{p,q}$ for the given power allocation variables $\left\{p_{R},p_{C}\right\}$. The goal of NSGA-II is to evolve this population over several generations such that the target variables converge to the global optimum. To this end, the evolutionary procedure of $e^{p,q}$ in each generation will be executed by the following rules:

- **Fitness**: The fitness is determined by the objective function in Equation (12), namely
  (15) $$f_{p,q}=\frac{\omega }{F_{R}}{\mathrm{ISLR}}^{-1}\left(e^{p,q}\right)+\frac{\left(1-\omega \right)}{F_{C}}R_{t}\left(e^{p,q}\right)$$
- **Selection**: The parent individuals are selected by the roulette strategy, where the selection probability of each individual is dominated by its fitness value.
- **Crossover**: Each individual is tantamount to a binary code. However, the binary encoding can not work well in the constrained evolution procedure because of the extra computation cost for determining whether the solution is feasible. To solve this, a projection-based space transformation method [38] is employed to convert the discrete binary feasible space into a continuous real-value feasible space.
- **Mutation**: To avoid the risk of premature convergence, a small percentage of children are required to mutate. The mutation operation is implemented in the above real-value feasible space. Then, the child and parent populations are merged to form an elite population [38].
- **Sorting and Ranking**: The feasible solutions are sorted based on the nondominated sorting approach. The dominance between any two individuals $e^{i,q}$ and $e^{j,q}$ is defined as: if ${\mathrm{ISLR}}^{-1}\left(e^{i,q}\right)>{\mathrm{ISLR}}^{-1}\left(e^{j,q}\right)$ while $R_{t}\left(e^{i,q}\right)>R_{t}\left(e^{j,q}\right)$, we say $e^{i,q}$ dominates $e^{j,q}$, and vice versa; otherwise, $e^{i,q}$ and $e^{j,q}$ are nondominant. Then, rank the dominances of all individuals from 1 to *P*, where rank 1 represents the individual that dominates all other individuals.

#### 3.2.2. Inner Tier for Power Allocation

The inner tier procedure for power allocation starts after the update of the population of the band selection variable e is completed in each generation. Since the power allocation variables $\left\{p_{R},p_{C}\right\}$ are not coupled with each other for a given e, the subproblem in (14) can be further partitioned into two power allocation sub-subproblems. Defining a matrix $\Gamma =\mathrm{diag}\left[e⊗1_{L}\right]\in R^{N_{s}\times N_{s}}$, the power allocation sub-subproblem for radar performance can be formulated as

(16a) $$\begin{array}{cc} \min_{p_{R}}\; & \alpha_{1}\sum_{\tau \in \Theta_{s}}{\left|f_{\tau }^{H}\Gamma p_{R}\right|}^{2} \end{array}$$

(16b) $$\begin{array}{cc} s.t. & 1_{N_{s}}^{T}\Gamma p_{R}=\rho \xi_{t} \end{array}$$

(16c) $$\begin{array}{c} {\left[p_{R}\right]}_{n}\geq 0,\;n=1,\ldots ,N_{s} \end{array}$$

and the power allocation sub-subproblem for communication performance is formulated as

(17a) $$\begin{array}{cc} \max_{p_{C}} & \alpha_{2}\sum_{k=1}^{K}\log_{2}\left(1+\left(1-e_{k}\right)g_{k}{\left[p_{C}\right]}_{k}\right) \end{array}$$

(17b) $$\begin{array}{cc} s.t. & \log_{2}\left(1+\left(1-e_{k}\right)g_{k}{\left[p_{C}\right]}_{k}\right)\geq \zeta ,\;k=1,\ldots ,K \end{array}$$

(17c) $$\begin{array}{cc} \; & \sum_{k=1}^{K}\left(1-e_{k}\right){\left[p_{C}\right]}_{k}=\left(1-\rho \right)\xi_{t} \end{array}$$

where $\alpha_{1}=\omega /F_{R}$ and $\alpha_{2}=\left(1-\omega \right)B_{C}/F_{C}$ are defined to simplify the formulations.

**Remark** **3.** *For sub-subproblem (16), it does not need to be formulated as the maximization of ${ISLR}^{-1}$ after the communication metric term is removed.*

*(1) For Radar Performance*: Let us consider the sub-subproblem (16) with respect to the power allocation variable $p_{R}$ for ISLR reduction. The sub-subproblem (16) can be transformed into the following quadratic optimization by introducing an auxiliary variable $\beta \sum_{l=-\Delta }^{-\Delta }{\left|f_{l}^{H}\Gamma p_{R}\right|}^{2}$, where β is set to be zero.

(18) $$\begin{array}{cc} \min_{p_{R}} & \alpha_{1}{∥V^{\frac{1}{2}}F_{N_{s}}^{H}\Gamma p_{R}∥}_{2}^{2}\\ s.t. & 1_{N_{s}}^{T}\Gamma p_{R}=\rho \xi_{t} \end{array}$$

where $V\in C^{2N_{s}\times 2N_{s}}$ is the diagonal matrix with elements

(19) $$V_{\tau \tau }=\left\{\begin{array}{c} \alpha_{1},\;\tau \in \left[-N_{s}+1,-\Delta \right)\cup \left(\Delta ,N_{s}\right]\\ \beta ,\;\;\tau \in \left[-\Delta ,\Delta \right] \end{array}\right.$$

From the term of $\Gamma p_{R}$, it is noteworthy that only $\eta N_{S}$ available elements of $p_{R}$ make a contribution to the ISLR minimization. Therefore, the optimization problem in Equation (18) should be revised as

(20) $$\begin{array}{cc} \min_{{\bar{p}}_{R}}\; & \alpha_{1}{∥V^{\frac{1}{2}}{\bar{F}}_{N_{s}}{\bar{p}}_{R}∥}_{2}^{2}\\ s.t.\; & 1_{\eta N_{s}}^{T}{\bar{p}}_{R}=\rho \xi_{t} \end{array}$$

where ${\bar{p}}_{R}=J\Gamma p_{R}\in R^{\eta N_{s}\times 1}$ stacks the corresponding $\eta N_{s}$ coefficients of $\Gamma p_{R}$, where J is the $\eta N_{s}\times N_{s}$ stack matrix with binary elements. Each row of J contains only one element equal to 1, whose position is selected by the nonzero element of band selection variable e. Likewise, ${\bar{F}}_{N_{s}}=JF_{N_{s}}\in R^{\eta N_{s}\times N_{s}}$ is obtained by extracting the corresponding rows from $F_{N_{s}}$.

The optimization problem in Equation (20) is a standard linear constrained quadratic programming (LCQP), which can be efficiently solved by the Lagrange multiplier method [36,39]. Thus, the closed-form solution of ${\bar{p}}_{R}$ is given by

(21) $${\bar{p}}_{R}=\rho \xi_{t}\frac{U^{-1}1_{\eta N_{s}}}{1_{\eta N_{s}}^{T}U^{-1}1_{\eta N_{s}}}$$

where U is a Herimitan positive semidefinite matrix, namely

(22) $$U={\bar{F}}_{N_{s}}V{{\bar{F}}^{H}}_{N_{s}}$$

*(2) For Communication Performance*: Now, we focus on the sub-subproblem (17) of optimal power allocation for the multiuser network. Since the dual problem of (17) is convex, it can be solvable under the Karush–Kuhn–Tucker (KKT) conditions [39]

(23a) $$\begin{array}{cc} \; & \gamma \ln2\left(1+\left(1-e_{k}\right)g_{k}{\left[p_{C}\right]}_{k}\right)-\left(1-e_{k}\right)g_{k}\left(\alpha_{2}+\eta_{k}\right)=0 \end{array}$$

(23b) $$\begin{array}{cc} \; & \gamma \left(\sum_{k=1}^{K}\left(1-e_{k}\right){\left[p_{C}\right]}_{k}-\left(1-\rho \right)\xi_{t}\right)=0 \end{array}$$

(23c) $$\begin{array}{cc} \; & \gamma_{k}\left[\zeta -\log_{2}\left(1+\left(1-e_{k}\right)g_{k}{\left[p_{C}\right]}_{k}\right)\right]=0 \end{array}$$

(23d) $$\begin{array}{cc} \; & \gamma_{k}\geq 0,\;k=1,\ldots ,K \end{array}$$

where γ and $\gamma_{k}$ for k=1,…,K represent Lagrange multipliers. Thus, the optimal power allocation $p_{C}$ for maximizing the sum-rate can be obtained by

(24) $${\left[p_{C}\right]}_{k}=\left\{\begin{array}{cc} 0, & e_{k}=1\\ \frac{1}{g_{k}}\left(2e^{\zeta }-1\right), & \frac{1}{g_{k}}>\vartheta ,e_{k}=0\\ \vartheta -\frac{1}{g_{k}},\; & \frac{1}{g_{k}}<\vartheta ,e_{k}=0 \end{array}\right.$$

where the positive parameter $\vartheta =\frac{1}{2g_{k}}e^{g_{k}\alpha_{2}-\gamma }$ can be obtained by a bisection search procedure when the γ meets the condition of

(25) $$\left(1-e_{k}\right)\sum_{k=1}^{K}\left(\vartheta -\frac{1}{g_{k}}\right)-\left(1-\rho \right)\xi_{t}=0$$

Once the optimal power allocation variables $\left\{p_{R},p_{C}\right\}$ are determined for each individual for the band selection variable e, the inner tier procedure is completed. $p_{R}$ and $p_{C}$ subsequently participate in the update of e in the outer tier procedure. These two procedures are alternately executed until convergency.

**Figure 3:**
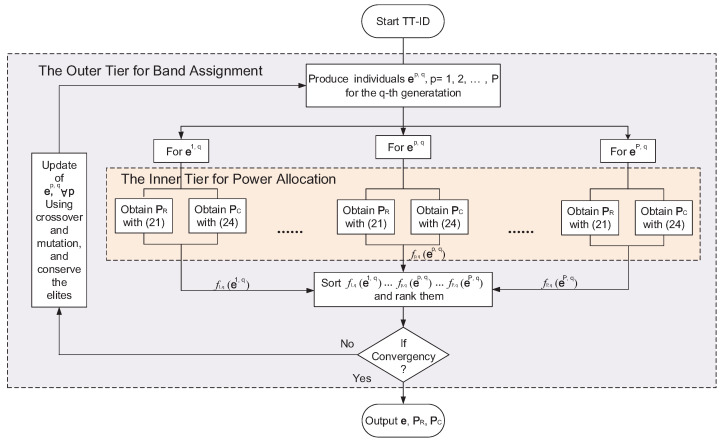
The flowchart of TT-ID algorithm.

The pseudocode of the bisection search of ϑ is given in Algorithm 1. Finally, the flowchart of the proposed TT-ID algorithm is given in Figure 3.
**Algorithm 1** Bisection search of ϑ.**Initialization:**$\vartheta_{\min}$, $\vartheta_{\max}$, t=0 and the tolerance δ>0
  1:**while**$\left|\left(1-e_{k}\right)\sum_{k=1}^{K}\left(\vartheta -\frac{1}{g_{k}}\right)-\left(1-\rho \right)\xi_{t}\right|>\delta$**do**  2:    **for** k=1,⋯,K **do**  3:          $\vartheta^{\left(t\right)}\leftarrow \left(\vartheta_{\min}+\vartheta_{\max}\right)/2$  4:          **if** $\left(1-e_{k}\right)\sum_{k=1}^{K}\left(\vartheta^{\left(t\right)}-\frac{1}{g_{k}}\right)>\left(1-\rho \right)\xi_{t}$ **then**  5:                $\vartheta_{\max}\leftarrow \vartheta^{\left(t\right)}$  6:                $\vartheta^{\left(t\right)}\leftarrow \left(\vartheta_{\min}+\vartheta_{\max}\right)/2$  7:          **else if** $\left(1-e_{k}\right)\sum_{k=1}^{K}\left(\vartheta^{\left(t\right)}-\frac{1}{g_{k}}\right)<\left(1-\rho \right)\xi_{t}$ **then**  8:                $\vartheta_{\min}\leftarrow \vartheta^{\left(t\right)}$  9:                $\vartheta^{\left(t\right)}\leftarrow \left(\vartheta_{\min}+\vartheta_{\max}\right)/2$  10:          **end if**  11:          Let t←t+1  12:    **end for**  13:**end while**

### 3.3. Complexity Analysis

The computational complexity of the TT-ID algorithm is dominated by the complexity per iteration. Considering the outer tier procedure for the band assignment subproblem (16), the complexity of the crossover and mutation in NSGA-II for *P* individuals is the order of $O\left(PK\right)$. Additionally, the complexity of sorting is of order $O\left(P\log_{2}P\right)$. In the inner tier procedure, for each individual, the computational complexity of the sub-subproblem (16) is dominated by matrix inversion, which is the order of $O\left(N_{s}^{3}\right)$. Besides, the complexity of the sub-subproblem (17) is $O\left(M\log_{2}\left(\vartheta_{\max}-\vartheta_{\min}\right)/\delta \right)$ due to the bisection search method, where *M* is the number of communication signals.

For comparison, the complexity analysis of two related works is provided. One of the related works in [40] aims to improve SINR and PSLR by assigning the adaptable bandwidths in the CRC system. To this end, two approaches are employed to solve the optimization problem, namely the weighted sum multiobjective optimization (WSMO) algorithm and NSGA-II algorithm. The complexity of WSMO is of order $O\left(K^{2}\right)$, while the complexity of NSGA-II for P individuals is of order $O\left(P\log_{2}2P\right)$ in each iteration. The other one in [41] proposes a joint subcarrier selection and power allocation scheme for maximizing the mutual information for radar and data rate for communication in a multicarrier dual-function radar-communication (DFRC) system. The convergence rate of the approach in [41] is dominated by the bisection search method, which is of order $O\left(M_{s}\log_{2}\left(\vartheta_{\max}-\vartheta_{\min}\right)/\delta \right)$, where $M_{s}$ denotes the number of subcarriers.

## 4. Sidelobe Reduction at Radar Receiver

In the last section, by allocating the adaptable working bands and transmit power for two subsystems, the sidelobes of the ACF of the radar signal can be reduced while guaranteeing a desired sum-rate for the multiuser network. However, compared with the conventional radar signals, the autocorrelation sidelobes of the resulting radar signal may still reach a high level due to its disjoint spectrum. Especially when the weighting factor ω tends to be 0, the sidelobe performance gets worse with high probability. To address this problem, in this section, we investigate how to further reduce the sidelobes at the radar receiver.

### 4.1. Signal Model at Radar Receiver

Let us consider the radar return signal at the radar receiver. The radar return signal can be viewed as the convolution of the transmitted waveform with the target responses of the observation area, namely

(26) $$x=b*\bar{z}+n$$

where $b\in C^{N_{t}\times 1}$ represents the $N_{t}$ impulse responses of the corresponding range cells in the observation area, $\bar{z}\in C^{N_{s}\times 1}$ denotes the temporal radar waveform, and $n\in C^{N_{p}\times 1}$ is the white Gaussian noise vector with the distribution of $N\left(0,\sigma_{n}^{2}\right)$. $N_{p}$ is the total length of the processing window, and it is not difficult to see that $N_{t}$, $N_{s}$, and $N_{p}$ satisfy the following identity

(27) $$N_{p}=N_{t}+N_{s}-1$$

Subsequently, the matched filtering procedure can be accomplished by

(28) $$\begin{array}{cc} y & ={\tilde{z}}^{r}*x\\ \; & ={\tilde{z}}^{r}*\left(b*\bar{z}+n\right) \end{array}$$

where ${\tilde{z}}^{r}$ refers to the complex conjugate and reverse of the temporal signal $\bar{z}$, which is the standard matched filter. According to the convolution rules, Equation (28) can be recast as

(29) $$\begin{array}{cc} y & =b*\left({\tilde{z}}^{r}*\bar{z}\right)+{\tilde{z}}^{r}*n\\ \; & =b*r+\bar{n} \end{array}$$

where $\bar{n}={\tilde{z}}^{r}*n\in C^{\left(N_{p}+N_{s}-1\right)\times 1}$ is the noise output of the matched filter.

Equation (29) suggests that the matched filter output y is regarded as the convolution of the ACF r of the probing waveform with the impulse response b of the scene, which explains why the target estimation performance of the matched filter highly depends on the correlation properties of the transmitted signal. It is not hard to imagine that the radar signal with intolerably high range sidelobes may result in a situation where the weak targets are most probably overshadowed by the neighboring strong targets.

### 4.2. Sidelobe Reduction Approach

As we mentioned before, the capacity of sidelobe reduction of the joint optimization is limited in some particular scenarios. Hence, in this subsection, our goal is to investigate a sidelobe reduction approach from the perspective of processing at the radar receiver.

The matched filtering procedure in Equation (29) can also be written as the frequency domain formulation according to the convolution rules, namely

(30) $$\begin{array}{cc} y & =F_{N_{y}}^{H}\left[\left(F_{N_{y}}^{1}b\right)⊙\left(F_{N_{y}}^{2}r\right)\right]+\bar{n}\\ \; & =F_{N_{y}}^{H}\left[\tilde{b}⊙\left(\Gamma p_{R}⊗1_{D}\right)\right]+\bar{n}\\ \; & =F_{N_{y}}^{H}\tilde{B}\left(\Gamma p_{R}⊗1_{D}\right)+\bar{n} \end{array}$$

where $F_{N_{y}}\in C^{N_{y}\times N_{y}}$, $F_{N_{y}}^{1}\in C^{N_{y}\times N_{p}}$, and $F_{N_{y}}^{2}\in C^{N_{y}\times 2N_{s}}$ denote the DFT matrices for the matched filtering, for the Fourier transform of the impulse response b, and for the Fourier transform of ACF r, respectively, where $N_{y}=DN_{s}\geq \left(N_{p}+N_{s}-1\right)$ for facilitating calculation, and the integer *D* satisfies

(31) $$D\geq \left\lceil \frac{N_{p}+N_{s}-1}{N_{s}}\right\rceil$$

$\tilde{B}=\mathrm{diag}\left[\tilde{b}\right]\in C^{N_{y}\times N_{y}}$ is a diagonal matrix, where the vector $\tilde{b}$ denotes the frequency domain target impulse response. Based on the time-frequency duality, it can be found that Equation (30) is similar to the classic spectral estimation problem for missing data in the frequency domain [42]. Like the definitions in Equation (20), we also introduce the stack matrix J to reconstruct the available samples vector $p_{r}\in R^{\eta N_{y}\times 1}$ and the corresponding DFT matrix ${\bar{F}}_{N_{y}}\in C^{\eta N_{y}\times N_{y}}$, namely

(32) $$p_{r}=\left(J⊗I_{D}\right)\tilde{B}\left(\Gamma p_{R}⊗1_{D}\right)$$

(33) $${\bar{F}}_{N_{y}}=\left(J⊗I_{D}\right)F_{N_{y}}$$

The objective function of spectral estimation with respect to the matched filter output y can then be formulated as

(34) $$\min_{y}\;\;{∥p_{r}-{\bar{F}}_{N_{y}}y∥}_{2}^{2}\;$$

However, the objective function in Equation (34) is a linear underdetermined problem, which can be possibly met by many feasible solutions. To obtain the unique solution, a regularized term is imposed to the objective function from Equation (34) as follows

(35) $$\min_{y}\;\;\Phi \left(y\right)+{∥p_{r}-{\bar{F}}_{N_{y}}y∥}_{2}^{2}\;$$

Obviously, the key to spectral estimation in Equation (35) is to select a reasonable regularizer $\Phi \left(y\right)$. As mentioned before, $\Phi \left(y\right)$ is in fact dominated by the ACF. It is well-known that a Gaussian-like shaped autocorrelation for the bandlimited signal is widely used as the ideal target spectra response, whose amplitude distribution with a high roll-off and a quite low sidelobe floor is “long-tailed” [14,15,43]. In particular, the Cauchy distribution, one of the Gaussian-like shaped functions, can further enhance the peaks and resolution of spectral amplitude [43]. Thus, we employ the Cauchy distribution to impose the regularizer, namely

(36) $$\Phi \left(y\right)=\sum_{l=1}^{N_{y}}\ln\left(1+\frac{y_{l}{y_{l}}^{*}}{2\sigma_{y}^{2}}\right)\;$$

where $\sigma_{y}$ denotes the scale parameter. Here, $\sigma_{y}$ is defined as the −3 dB mainlobe width (i.e., range resolution) of the ACF of the radar waveform z. In practice, the −3 dB mainlobe width can be estimated by calculating the reciprocal of the root-mean-square bandwidth of the desired PSD without spectral nulls [35]. Therefore, $\sigma_{y}$ can be preset at the beginning of the spectral estimation algorithm.

Substituting Equation (36) into Equation (35), taking the derivative of Equation (35) and letting it equal to zero, the optimal solution to the objective function in Equation (35) is obtained by

(37) $$y=Q{\bar{F}}_{N_{y}}{\left(\lambda I_{N_{y}}+{\bar{F}}_{N_{y}}Q{\bar{F}}_{N_{y}}^{H}\right)}^{-1}p_{r}\;$$

where $\lambda =\sigma_{n}^{2}/\sigma_{y}^{2}$ is the ridge regression parameter. $Q\in C^{N_{y}\times N_{y}}$ is the diagonal matrix with entries

(38) $$Q_{ll}=1+\frac{y_{l}{y_{l}}^{*}}{2\sigma_{y}^{2}},\;l=1,\ldots ,N_{y}$$

Because of the Cauchy and Gaussian distribution models for target response and noise, respectively, established, as well as the Bayes theorem applied in this spectral estimation algorithm, we refer to it as the Bayes–CG algorithm.

From Equations (37) and (38), it is noteworthy that the matrix Q is determined by y, which suggests that the solution from Equation (37) has to be computed by an iterative procedure. The iterative procedure is provided as follows.

Letting $d={\left(\lambda I_{N_{y}}+{\bar{F}}_{N_{y}}Q{\bar{F}}_{N_{y}}^{H}\right)}^{-1}p_{r}$, the solution in Equation (37) can be rewritten as

(39) $$y=Q{\bar{F}}_{N_{y}}d$$

Accordingly, we first initialize y using the matched filter output in Equation (30). Next, in the *t*-th iteration, we compute the $d^{\left(t\right)}$ as

(40) $$d^{\left(t\right)}={\left[\lambda I_{N_{y}}+{\bar{F}}_{N_{y}}Q^{\left(t\right)}{{\bar{F}}_{N_{y}}}^{H}\right]}^{-1}p_{r}$$

Then, update the y, namely

(41) $$y^{\left(t+1\right)}=Q^{\left(t\right)}{\bar{F}}_{N_{y}}d^{\left(t\right)}$$

The iterative procedure is terminated until the following condition is reached

(42) $${∥y^{\left(t+1\right)}-y^{\left(t\right)}∥}_{2}^{2}\leq \varepsilon \;$$

where ε represents the difference tolerance between two successive iterations.

The iterative procedure of the Bayes–CG algorithm to minimize the objective function in Equation (35) is listed in Algorithm  2, and we refer to Appendix A to know the detailed derivations of Equation (37).

**Remark** **4.** *The basis of the spectral estimation algorithm above is to recover missing samples (i.e., spectral nulls) by using interpolation and extrapolation based on the received available data (i.e., $p_{r}$) and the prior distribution of the desired autocorrelation response. Therefore, it can be seen that the nulls in $\Gamma p_{R}$ will be filled using the DFT of the optimal estimator y.*

**Algorithm 2** Bayes–CG algorithm for solving Equation (35)**Initialization:**$y^{\left(0\right)}$, t=0, and ε>0
  1:
**while** ${∥y^{\left(t+1\right)}-y^{\left(t\right)}∥}_{2}^{2}>\varepsilon$ **do**
  2:    Compute the $d^{\left(t\right)}$ according to Equation (40)  3:    Update the $y^{\left(t\right)}$ using Equations (40) and (41)  4:    Let t←t+1  5:
**end while**


**Output:**

$y^{*}$



## 5. Numerical Results

In this section, we provide various numerical results to verify the effectiveness of the proposed joint resource allocation optimization. The resulting radar waveform is synthesized by PSD fitting in [12], and quadrature amplitude modulation (QAM) signals are used for data delivery. We assume that the frequency response of the communications channel is determined by the standard normal distribution, and the noise is complex additive white Gaussian noise. The other simulation parameters are given in Table 1.

We denote the proposed joint band assignment and power allocation scheme as “JBAPA” and employ the following four resource allocation schemes to form a comparison:

- *Random Band Assignment and Equal Power Allocation (RBAEPA)*: The working bands are randomly assigned to both functions. In each subsystem, the transmit power allocated on the corresponding scale frequency bin is uniformly distributed.
- *Random Band Assignment and Optimal Power Allocation (RBAPA)*: The working bands are randomly assigned to both functions, whereas the transmit power allocations for two functions are implemented by the KKT conditions, respectively.
- *Optimal Band Assignment and Equal Power allocation (BAEPA)*: The best working band assignment is optimized by NSGA-II, while the PSD distribution of radar waveform is a rectangle shape, and transmit powers for communication users are equal to each other.

### 5.1. Performance Comparision

**Figure 4:**
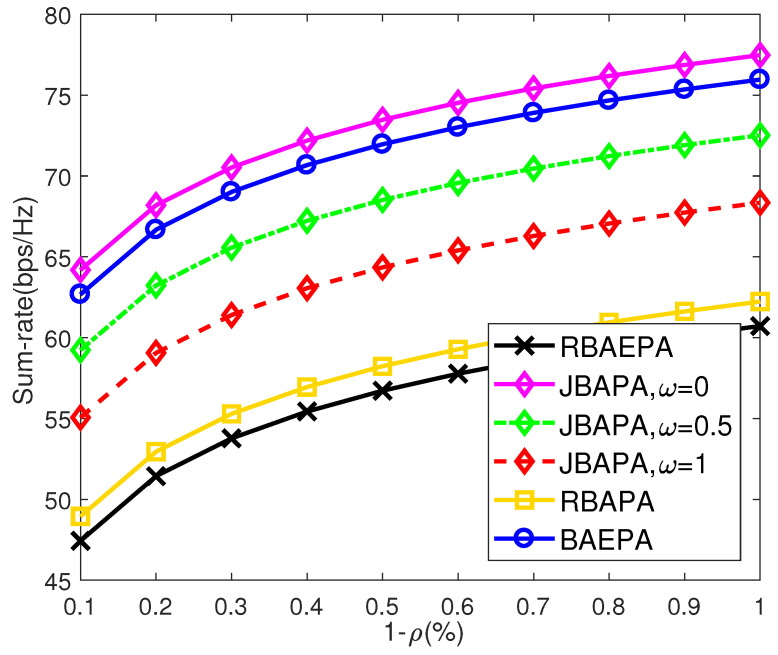
Comparison of the sum-rate of different schemes, where η=75%.

Under different resource allocation schemes, the sum-rate performance with the power occupancy ratio (i.e., 1−ρ) of the communication subsystem is depicted in Figure 4. Here, each communication link is assumed to occupy an 8 MHz bandwidth, namely the bandwidth occupancy ratio η=75%. From Figure 4, we can see that by occupying more system power, the sum-rate performance is enhanced, and the proposed JBAPA scheme outperforms most of the resource allocation schemes, even if the weighting factor ω equals 1. That is because the JBAPA scheme makes full use of two-dimensional bandwidth-power resources to enhance the sum-rate performance, whereas the RBAPA and BAEPA schemes only employ the DOF of a one-dimensional resource, which causes limited improvement. Furthermore, compared with the RBAEPA scheme, the other three schemes validate the benefits of band assignment and power allocation for CRC systems.

**Figure 5:**
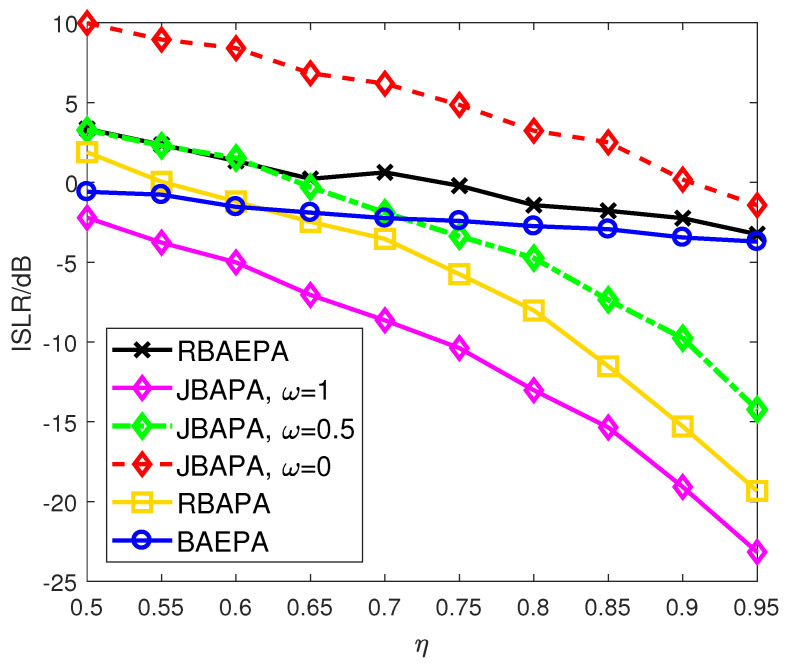
Comparison of the ISLR of different schemes.

Similarly, compared with other resource allocation schemes, the outstanding ISLR performance of the proposed JBAPA scheme in most cases is shown in Figure 5. As expected, the autocorrelation sidelobe level of the transmit waveform gets better with the increase of η. That is because more resources can be utilized as the degrees of freedom to achieve radar waveform optimization. However, when the bandwidth occupancy ratio η is in the interval (0.5, 0.65) and the weighting factor ω tends to a smaller value, it is shown that the ISLR of the JABAPA scheme rises to an unacceptably high level in most cases, not to mention the other schemes. That is why we further investigate a sidelobe reduction method at the radar receiver. Besides, by comparing the RBAPA scheme with the BAEPA scheme, it reveals that the power allocation can make greater contributions to ISLR minimization than the band assignment unless a few bandwidth resources are allocated to radar subsystem.

**Figure 6:**
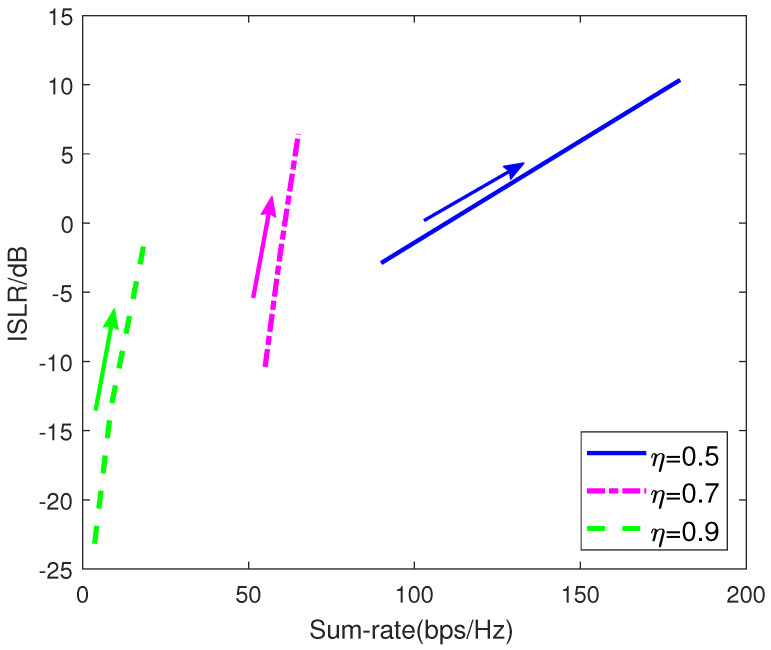
The optimal trade-off curve.

Figure 6 plots the optimal tradeoff curve of radar performance and communication performance. The weighting factor ω for the radar is decreasing from 1 to 0 along the direction of the arrow. The ISLR performance suffers from the degradation with the ω decreasing, whereas the sum-rate is improved. According to the above results, the control center in the proposed CRC system will select the appropriate weighting factor and power occupancy ratio to meet the practical demands for the ISLR and sum-rate.

### 5.2. Sidelobe Reduction at Radar Receiver

As mentioned above, in this subsection, we study the capacity of range sidelobe suppression through the proposed spectral estimation algorithm at radar receiver.

Consider a scenario where the best four channels have been assigned to corresponding communication links to maximize the sum-rate (i.e., ω=0). We assume that there are 20 targets randomly located at the range from 3500 m to 5500 m in the observing area, whose SNRs are also randomly assigned subject to a uniform distribution on the interval (15, 80) dB.The ground truth involving the zero-mean additive complex Gaussian noise is provided as the baseline. With the loss of generality, we here assume that the Doppler shift of the target has been precisely estimated and compensated before the matched filtering.

**Figure 7:**
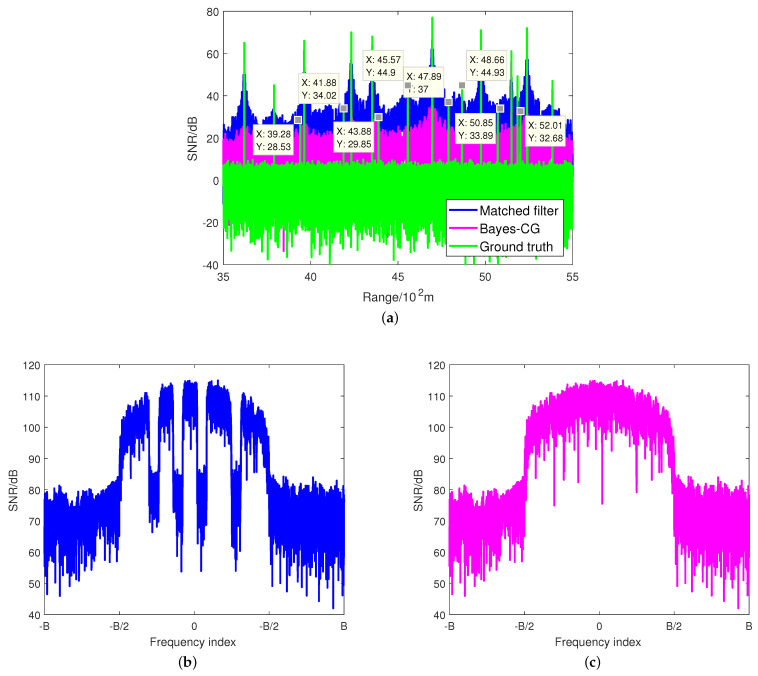
(**a**) Target estimation comparison between matched filtering and Bayes–CG algorithm, where η=75%. (**b**) PSD of the target estimation result using a standard matched filter; (**c**) PSD of the target estimation result using the Bayes–CG algorithm.

The target estimation results of the matched filtering and the proposed Bayes–CG algorithm are shown in Figure 7a. As predicted, the matched filtering results in an extremely high sidelobe level due to the poor ISLR performance caused in the radar’s worst case. In particular, it can be seen that the targets in (3928, 4188, 4388, 5201) m, which are overshadowed by the neighboring higher SNR target, respectively, fail to be detected through the matched filtering. Additionally, the SNRs of the targets located at (4557, 4789, 5085) m are comparable with the sidelobe level of the nearby strong targets, which may make the radar confused possibly. On the other hand, the Bayes–CG algorithm provides a great target estimation performance. All of the targets can be detected significantly because the average sidelobe level of the Bayes–CG algorithm is 40 dB lower than the level of the matched filter. Furthermore, Figure 7b,c plot the PSDs of the target estimation result using matched filter and Bayes–CG methods, respectively. It is noticed that the existing spectral nulls during the matched filtering have been filled in after the implementation of the Bayes–CG algorithm. Note that it is similar to the PSD of the generalized nonlinear frequency modulation (NLFM) waveform, whose sidelobe level can reach −40 dB below.

Generally speaking, the performance of the spectral estimation algorithm may degrade in high data missing ratio cases. In what follows, the cases of a high bandwidth occupancy ratio η compared with the missing ratio are taken into consideration. To show the robust performance of the presented spectral estimation algorithm under varying ρ conditions, we compare it with the normalized matched filter and the following three typical sidelobe reduction approaches from the perspective of processing:

- *Least-Square (LS) Filter*: This approach is a well-known method to reduce the range sidelobe for arbitrary modulation signals.
- *Spectral Nulls Oriented (SNO) Mismatched Filter* [44]: The mismatched filter is specially designed for ISLR minimization of spectrally compliant waveforms. In such a method, the filter response can achieve the same range sidelobe level as the that of the transmit waveform without frequency notches based on the concept of the inverse filter.
- *Autoregressive (AR) Based Interpolation* [45]: A straightforward method to solve the missing sample problem is to interpolate the gaps between the disjoint samples. The AR coefficients derived by the Burg algorithm are used to achieve the interpolation, which is denoted as the AR-Burg approach. It has been widely used for high-resolution imaging in an interrupted synthetic aperture radar (SAR).

Here, we consider a scenario containing only one target to focus on the resulting range sidelobe level after applying the aforementioned methods. Here, we are more concerned about the normalized peak sidelobe level (PSL), which is defined as

(43) $$\mathrm{PSL}=20\log_{10}\frac{\max_{l\in \Theta_{s}}\;\left|r_{l}\right|}{\left|r_{0}\right|}$$

Moreover, we employ 100 standard Monte Carlo trails to guarantee the reliability of simulations.

**Figure 8:**
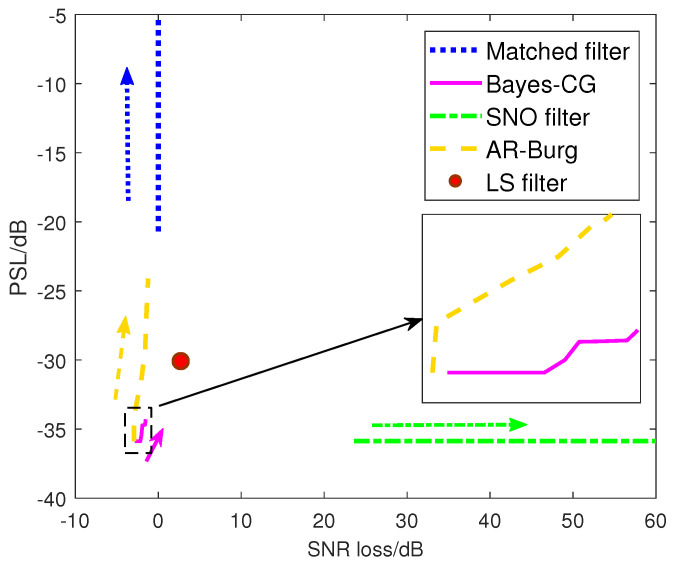
PSL versus SNR loss under different sidelobe reduction approaches with the decreasing bandwidth occupancy ratio η, where ω=0.

Figure 8 provides the PSL and SNR loss of different approaches under varying bandwidth occupancy ratio η conditions. The direction of the arrow indicates η is decreasing from 95% to 50%. From Figure 8, it can be seen that the four approaches can suppress the range sidelobes effectively when the η is large, even the matched filter. As η decreases, however, the PSL of the matched filter output rapidly rises to approximately −5 dB, which results in the radar hardly detecting the weak targets from the interferences of the nearby strong targets. The performances of the AR-Burg approach and SNO approach also suffer different degrees of degradation. Note that the PSL of the AR-Burg approach is increasing significantly with the decrease of η due to the unsatisfied demand for sufficient available samples. Nevertheless, the SNR may slightly benefit from the AR-Burg approach because the data gaps are compensated by interpolation and extrapolation. In contrast, although the SNO approach can achieve the same sidelobe as NLFM waveform (e.g., −40 dB below), similar to the conventional inverse filter, the SNR loss is very sensitive to both spectral null depth and the bandwidth occupancy ratio due to the fact that the power of received noises embed in nulls are significantly amplified during processing. The LS filter is a relatively robust approach, which always maintains about −30 dB of PSL unchanged with a slight SNR loss, due to the fact that the capacity of sidelobe suppression of the LS filter is only dependent on the filter length. Compared with the above approaches, the outstanding robust performance of the Bayes–CG algorithm is shown. Moreover, it still behaves well when the η decreases to 50%, i.e., the performance loss of PSL does not exceed 3 dB. That is because compared with the AR-Burg-based interpolation, the Bayes–CG algorithm makes the best of the prior knowledge of the autocorrelation response instead of partial information, which guarantees the desired sidelobe level with a fine SNR gain.

### 5.3. Algorithm Convergence

In this subsection, the convergences of the proposed TT-ID algorithm and Bayes–CG algorithm are evaluated. For TT-ID, the variation of relative change between any two consecutive generations is employed as the convergence criteria, which are given by

(44) $$R_{ch}=\frac{\left|f_{p+1}^{{}^{\prime }}-f_{p}^{{}^{\prime }}\right|}{\left|f_{p+1}^{{}^{\prime }}\right|}$$

where $f_{p}^{{}^{\prime }}$ denotes the local optimum of the p-th generation. For the Bayes–CG algorithm, the relative change metric is redefined as

(45) $$R_{ch}={∥y^{\left(t+1\right)}-y^{\left(t\right)}∥}_{2}^{2}$$

**Figure 9:**
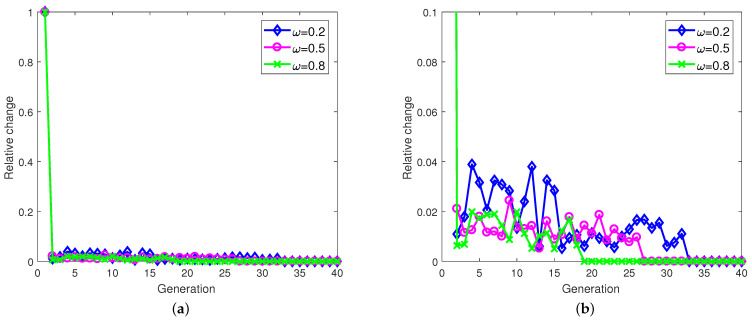
The convergence of the proposed TT-ID algorithm, where η=75%. (**a**) Completed; (**b**) Part enlarged.

**Figure 10:**
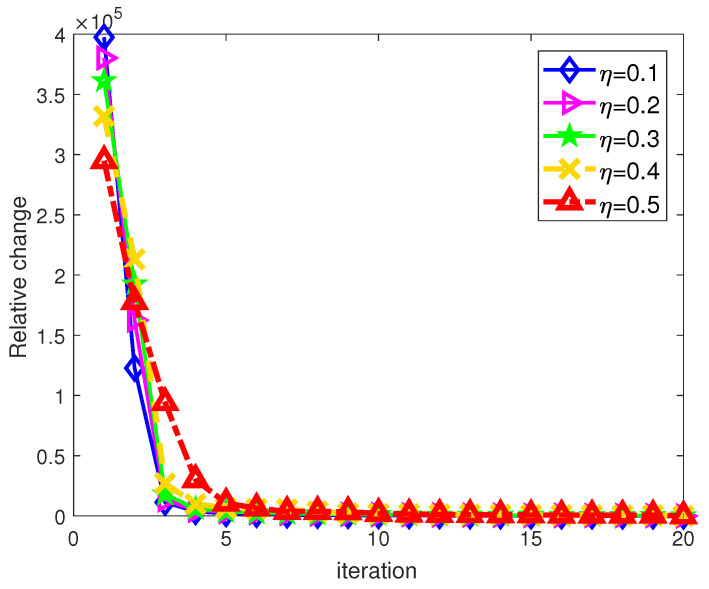
The convergence of the Bayes–CG algorithm.

In Figure 9, it can be seen that using the TT-ID algorithm, the objective functions in Equation (12) under different weighting factor conditions are converged within the 20th to 35th generation, where η is set to be 75%. Moreover, only about 10 iterations are spent on target spectra estimation through the Bayes–CG algorithm under different η, as shown in Figure 10. These numerical results reveal that our proposed approaches can effectively address the joint resource allocation and sidelobe reduction problem at the radar receiver, respectively, within a few tens of iterations.

## 6. Conclusions

In this paper, we propose a joint optimization framework for a frequency-division-based CRC system. In the proposed CRC system, the spectrally compliant waveform is used for target detection, and communication signals serve the multiuser network via the FDM technique. Moreover, a control center plays a key role in implementing optimal resource management, which can force the cooperation between the radar and communication subsystems. By taking the minimization of ISLR for target detection and the maximization of the sum-rate for the multiuser communication network into account, a joint band assignment and power allocation strategy with some relevant requirements is a multiobjective nonconvex optimization problem, which is solved iteratively by using decomposition and global minimization. However, the autocorrelation sidelobes of the resulting radar signal may still maintain a relatively high level compared with the conventional radar signals, especially when the data transmission is the primary purpose. By deriving the signal model at the radar receiver, the target detection through the matched filtering can be viewed as a spectral estimation problem of missing samples. Thus, a Bayes–CG algorithm is employed to further suppress the range sidelobes of the matched filter output to a quite low floor. Simulations and theoretical analysis have shown that the proposed scheme outperforms the conventional schemes in the CRC system, and the presented ISLR minimization based on the spectral estimation algorithm is effective and robust in the radar’s worst case.

Since the proposed TS-HD algorithm suffers from a high computational complexity, future studies will investigate a more efficient approach to solve the joint resource allocation problem for the CRC system. Moreover, it is worthy to note that the range resolution may not maintain a great condition, particularly in some cases. Hence, the range resolution will be taken into account in our future work. 

## Figures and Tables

**Table 1:** Simulation parameters.

Parameter	Value	Parameter	Value
Total bandwidth *B*	128 MHz	Center frequency $f_{c}$	5 GHz
Total power $\xi_{t}$	1000 W	Noise power $\sigma_{C}$	$1\times 10^{-14}$ W/Hz
Communication users *M*	4	Waveform sample length $N_{s}$	512
Generation *Q*	100	Population *P*	100
Crossover probability	0.9	Mutation probability	0.1

## Appendix A

First of all, we explain how to use the Cauchy distribution to impose the regularizer in Equation (36).

Without loss of generality, it is assumed that the radar transmit waveform $\bar{z}$ satisfies $\left|\bar{z}\right|=1$. Hence, the output noise $\bar{n}$ is still subject to $N\left(0,{\sigma_{n}}^{2}\right)$ after the matched filtering. Based on Bayes’ theorem, we can obtain the conditional distribution of the $p_{r}$ as

(A1) $$p\left(p_{r}\left|y,\sigma_{n}\right.\right)={\left(\frac{1}{2\pi \sigma_{n}^{2}}\right)}^{N_{y}}\exp\left(-\frac{1}{2\sigma_{n}^{2}}{∥p_{r}-{\bar{F}}_{N_{y}}^{H}y∥}_{2}^{2}\right)\;$$

Furthermore, the *a posterior* distribution of the y is formulated as

(A2) $$p\left(y\left|p_{r},\sigma_{y},\sigma_{n}\right.\right)=\frac{p\left(y\left|\sigma_{y}\right.\right)p\left(p_{r}\left|y,\sigma_{n}\right.\right)}{p\left(p_{r}\left|\sigma_{y},\sigma_{n}\right.\right)}\;$$

The $p\left(y\left|p_{r},\sigma_{y},\sigma_{n}\right.\right)$ can be maximized using the maximum *a posterior* (MAP) estimator, for the given $\sigma_{y}$ and $\sigma_{n}$. Since the desired y approximates the Cauchy distribution, it can be modeled as

(A3) $$p\left(y\;\left|\;\sigma_{y}\right.\right)\propto \underset{N_{y}}{\Pi }\;{\left(1+\frac{y_{l}y_{l}^{*}}{2\sigma_{y}^{2}}\right)}^{-1}\;$$

Accordingly, the desired regularizer is obtained in Equation (36).

Then, let us consider the derivatives of the objective function in Equation (35). It is not difficult to compute the derivative of the second part in Equation (35), namely

(A4) $$\frac{\partial }{\partial y^{*}}{∥p_{r}-{\bar{F}}_{N_{y}}^{H}y∥}_{2}^{2}=\frac{1}{2\sigma_{n}^{2}}{\bar{F}}_{N_{y}}\left(p_{r}-{\bar{F}}_{N_{y}}^{H}y\right)$$

Subsequently, we focus on the derivative of the regularization term

(A5) $$\begin{array}{cc} \frac{\partial }{\partial {y_{l}}^{*}}\Phi \left(y\right) & =\frac{\partial }{\partial {y_{l}}^{*}}\sum_{l=1}^{N_{y}}\ln\left(1+\frac{y_{l}{y_{l}}^{*}}{2\sigma_{y}^{2}}\right)\\ \; & =\frac{1}{2\sigma_{y}^{2}}{\left(1+\frac{y_{l}{y_{l}}^{*}}{2\sigma_{y}^{2}}\right)}^{-1}y_{l}\\ \; & =\frac{1}{2\sigma_{y}^{2}}{Q_{ll}}^{-1}y_{l} \end{array}$$

Transform (A5) into the matrix form, namely

(A6) $$\frac{\partial }{\partial y^{*}}\Phi \left(y\right)=\frac{1}{2\sigma_{y}^{2}}Q^{-1}y$$

Combining Equations (A4) and (A6) and letting it equal to zero, we have

(A7) $$y={\left(\lambda Q^{-1}+{\bar{F}}_{N_{y}}{\bar{F}}_{N_{y}}^{H}\right)}^{-1}{\bar{F}}_{N_{y}}p_{r}$$

Note that the Equation (37) is tantamount to Equation (A7) by applying the following transformations, and the former is more suitable to use for the iterative computations

(A8) $${{\bar{F}}_{N_{p}}}^{H}\left(\lambda I_{N_{y}}+{\bar{F}}_{N_{y}}Q{\bar{F}}_{N_{y}}^{H}\right)=\left(\lambda Q^{-1}+{\bar{F}}_{N_{y}}{\bar{F}}_{N_{y}}^{H}\right)Q{\bar{F}}_{N_{y}}$$

(A9) $${{\bar{F}}_{N_{y}}}^{H}{\left(\lambda Q^{-1}+{\bar{F}}_{N_{y}}{\bar{F}}_{N_{y}}^{H}\right)}^{-1}=Q{\bar{F}}_{N_{y}}{\left(\lambda I_{N_{y}}+{\bar{F}}_{N_{y}}Q{\bar{F}}_{N_{y}}^{H}\right)}^{-1}$$

where the terms $\left(\lambda Q^{-1}+{\bar{F}}_{N_{y}}{\bar{F}}_{N_{y}}^{H}\right)$ and $\left(\lambda I_{N_{y}}+{\bar{F}}_{N_{y}}Q{\bar{F}}_{N_{y}}^{H}\right)$ are both positive definite.
